# The Effect of the Material Periodic Structure on Free Vibrations of Thin Plates with Different Boundary Conditions

**DOI:** 10.3390/ma15165623

**Published:** 2022-08-16

**Authors:** Jarosław Jędrysiak

**Affiliations:** Department of Structural Mechanics, Łódź University of Technology, al. Politechniki 6, 90-924 Łódź, Poland; jarek@p.lodz.pl

**Keywords:** periodic plates, effect of microstructure, tolerance modelling, free vibrations

## Abstract

Thin elastic periodic plates are considered in this paper. Since the plates have a microstructure, the effect of its size on behaviour of the plates can play a crucial role. To take into account this effect, the tolerance modelling method is applied. This method allows us to obtain model equations with constant coefficients, which involve terms dependent of the microstructure size. Using the model equations, not only can formulas of fundamental lower-order vibration frequencies be obtained, but also formulas of higher-order vibration frequencies related to the microstructure. In this paper, the effect of the material periodic microstructure on free vibration frequencies for various boundary conditions of the plates was analysed. To obtain proper formulas of frequencies, the Ritz method is applied. Moreover, some results are compared to the results calculated using the FEM.

## 1. Introduction

**Figure 1:**
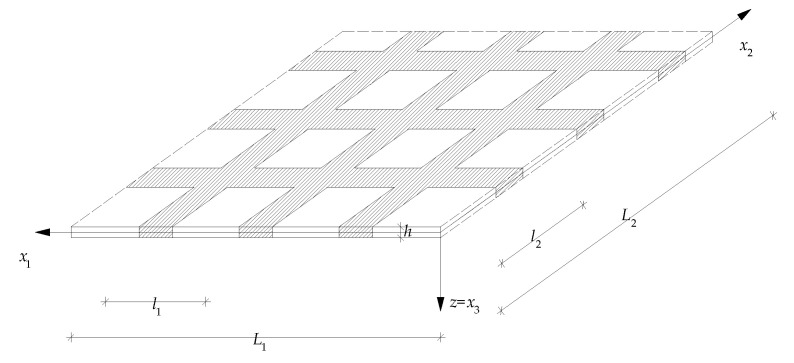
A fragment of a thin periodic plate.

In this paper, thin elastic plates with a periodic structure in planes parallel to the plate midplane are the main object under consideration. Plates of this kind are made of many identical repeatable elements, which are called *periodicity cells* (marked by dotted lines in Figure 1). Problems of vibrations of these plates can be analysed using partial differential equations, having highly oscillating, periodic, and non-continuous functional coefficients. Because these equations are not good tools to consider special examples of these plates, various simplified approaches are proposed. These methods make it possible to obtain governing equations with constant coefficients. Between them, those based on the asymptotic homogenization should be mentioned, cf. [1]. However, the effect of the microstructure size is usually omitted in the model governing equations.

In order to model various mechanical problems of periodic (or microheterogeneous) structures and composites, other methods are also used. The microlocal parameters approach was applied to describe such plates, e.g., in [2]. In the paper [3], honeycomb sandwich composite shells were analysed using the two-scale asymptotic homogenization method. Euler–Bernoulli beams were considered in [4], where the relationship between the 3D and the homogenisation approach was presented. The stability of multi-cell thin-walled columns of rectangular cross-sections was analysed theoretically, numerically, and experimentally in the work [5]. A dynamic critical load for buckling of columns was considered in [6]. Global and local buckling of sandwich beams and plates were investigated in [7]. A finite element unit-cell method was used in [8] to analyse heterogeneous plates. Brito-Santana et al. [9] applied an asymptotic dispersive method to the problem of shear-wave propagation in a laminated composite. Analytical and numerical methods were used to analyse buckling problems for sandwich beams with variable properties of the core in papers [10,11]. For laminated composited plates, various approaches were applied, e.g., the strong formulation isogeometric analysis in the work [12]; a layer-wise theory combined with a differential quadrature method in [13]. A generalized differential quadrature method was also used for vibrations of nanocomposite functionally graded shells in a series of papers [14,15,16]. An analytical-numerical model based on analytical relations and finite element method was shown to analyse a torsion of auxetic composite beams with a cellular structure in [17].

Unfortunately, the proposed modelling methods for microstructured periodic media usually lead to the model equations without the effect of the microstructure size. On the other side, this effect can play a crucial role in dynamical problems of these media, cf. Brillouin [18], where there were observed macro- and microvibrations related to the macro- and microstructure, respectively. Some special methods were adopted to analyse some similar problems to describe this effect. For instance, a spectral element method was proposed to investigate vibration band gap of periodic Mindlin’s plates in [19]. The centre finite difference method was used in [20] to analyse free flexural vibrations of periodic stiffened thin plates. The simplified super element method was applied for these plates filled with viscoelastic damping material in [21]. Problems of wave bandgaps in periodic plate structures were considered using differential quadrature element in [22].

An alternative approach to analyse various mechanical problems of microstructured (periodic and non-periodic) media is *the tolerance modelling method* (or *the tolerance method*), cf. the books Woźniak and Wierzbicki [23], Woźniak et al. (eds.) [24]. This method is general and useful for modelling different problems, which are described by differential equations with highly oscillating, non-continuous functional coefficients. It leads from these exact governing equations to the averaged model equations, which have constant (or slowly-varying) coefficients, some of which explicitly are dependent of the size of the microstructure.

Various applications of this method for different periodic structures were shown in a series of papers. Dynamics of some plane periodic structures were analysed in [25]. Vibrations of one-directional periodic plates, having thickness smaller than the length of the periodic cell, were considered in [26]. Vibrations of periodic wavy-type plates were investigated in [27]. Dynamics of thin plates reinforced by a system of periodic stiffeners was presented in [28]. Vibrations of medium thickness periodic plates were considered in [29]. The buckling of periodic plates interacting with a periodic elastic foundation was investigated in [30]. Vibrations of thin periodic plates with the microstructure size of an order of the plate thickness were described in [31]. Stability of periodic shells was considered in papers [32,33]. Dynamics problems of medium thickness plates on a periodic foundation were described in [34]. Geometrically nonlinear thin periodic plates were considered in [35]. Vibration analysis of periodic geometrically nonlinear beams is shown in [36]. Modelling of vibrations of periodic three-layered plate-type structures were investigated in [37]. Revisiting version of the modelling for dynamics and stability for periodic slender visco-elastic beams on a foundation with damping was shown in [38]. A multi-scale analysis of stress distribution in thin composite periodic plates was considered in [39].

Moreover, the tolerance method was also successfully used to analyse structures with non-periodic micro-heterogeneity, which can be treated as being made of functionally graded materials in the macro-scale. For instance, dynamics for longitudinally graded plates was investigated in [40,41], but stability of similar plates in [42]. Modelling of thin-walled structures with dense system of ribs was shown in [43,44]. Heat conduction of composite cylindrical conductors with non-uniform distribution of constituents was analysed in [45,46]. Effect of microstructure in problems of thermoelasticity for transversally graded laminates was considered in [47]. Dynamics of thin functionally graded plates with one-directional microstructure was investigated in [48]. Free vibrations of medium thickness functionally graded plates were analysed in [49]. Dynamics of thin functionally graded microstructured shells was investigated in [50,51], but dynamics and stability of them in [52]. Stability problems of functionally graded microstructured thin plates on elastic foundation were considered in [53]. However, the above-mentioned works do not cover all the problems that were studied by these authors in the literature on the tolerance modelling method.

The main aim of this contribution is to apply *the tolerance* and *asymptotic models of dynamic problems for thin elastic periodic plates*, cf. [26,30], to free vibrations of periodic plate strips with various boundary conditions. The other aim is a certain analysis of the effect of the material periodic cell structure on free vibration frequencies, formulas of them are derived using the Ritz method. Some results are compared and justified by the finite element method.

## 2. Modelling Foundations

### 2.1. Preliminaries

Let 0*x*_1_*x*_2_*x*_3_ denote the orthogonal Cartesian co-ordinate system in the physical space and *t* be the time coordinate. Subscripts α, β, …(*i*, *j*, …) run over 1, 2 (1, 2, 3) and indices *A*, *B*, … (*a*, *b*, …) run over 1, …, *N* (1, …, *n*). Summation convention holds for all aforementioned indices. Denote also **x** ≡ (*x*_1_,*x*_2_) and *z* ≡ *x*_3_. Let the region Ω ≡ {(**x**,*z*): −*h*(**x**)/2 < *z* < *h*(**x**)/2, **x** ∈ Π} be the undeformed plate, with Π as the midplane, having along the *x*_1_- and *x*_2_-axis length dimensions *L*_1_ and *L*_2_, respectively; and *h*(**x**) as the plate thickness.

Plates under consideration are assumed to be periodic in planes parallel to the plate midplane, with periods *l*_1_ and *l*_2_ along the *x*_1_- and *x*_2_-axis directions, respectively. Let ∆ ≡ [−*l*_1_/2, *l*_1_/2] × [−*l*_2_/2, *l*_2_/2] denote the periodicity basic cell on 0*x*_1_*x*_2_ plane. The cell size can be described by a parameter *l* ≡ [(*l*_1_)^2^ + (*l*_2_)^2^]^1/2^, which satisfies the condition max(*h*) < *l* << min(*L*_1_,*L*_2_), and can be called *the microstructure parameter*. Denote by (∙), _α_ ≡ ∂/∂*x*_α_ the partial derivatives with respect to the space co-ordinate, and by the overdots the partial derivatives with respect to the time co-ordinate.

It is assumed that all plate properties can be periodic functions in **x**, e.g., thickness *h*(**x**), elastic moduli *a_ijkl_* = *a_ijkl_*(**x**,*z*), mass density ρ = ρ(**x**,*z*). Moreover, these plate material properties are even functions in *z*. By *a*_αβγδ_, *a*_αβ33_, *a*_3333_ denote the non-zero components of the elastic moduli tensor, and also introduce: *c*_αβγδ_ ≡ *a*_αβγδ_ − *a*_αβ33_*a*_γδ33_(*a*_3333_)^−1^.

Let *u_i_*, *e_ij_* and *s_ij_* denote displacements, strains, and stresses, respectively; and ${\bar{u}}_{i}$ and ${\bar{e}}_{ij}$—virtual displacements and virtual strains; and *p*—loadings (along the *z*-axis).

Considerations are based on the well-known assumptions of the Kirchhoff-type plate theory, but they are mentioned below.

- *The kinematic assumptions*

(1) $$u_{\alpha }(x,z,t)=-z\partial_{\alpha }w(x,t),\;u_{3}(x,z,t)=w(x,t),$$

where *w*(**x**,*t*) is the deflection of the midplane. Similar assumptions are made for virtual displacements:

(2) $${\bar{u}}_{\alpha }(x,z)=-z\partial_{\alpha }\bar{w}(x),\;{\bar{u}}_{3}(x,z)=\bar{w}(x).$$

- *The strain-displacement relations*

(3) $$e_{\alpha \beta }=u_{\left(\alpha ,\beta \right)}.$$

- *The stress-strain relations*

(with the assumption that the plane of elastic symmetry is parallel to the plane *z* = 0)

(4) $$s_{\alpha \beta }=c_{\alpha \beta \gamma \delta }e_{\gamma \delta },$$

where:

(5) $$c_{\alpha \beta \gamma \delta }=a_{\alpha \beta \gamma \delta }-a_{\alpha \beta 33}a_{33\gamma \delta }/a_{3333},\;c_{\alpha 3\gamma 3}=a_{\alpha 3\gamma 3}-a_{\alpha 333}a_{33\gamma 3}/a_{3333}.$$

- *The virtual work equation*
(6) $$\int_{\Pi }\int_{-h/2}^{h/2}\rho {\ddot{u}}_{i}{\bar{u}}_{i}dzda+\int_{\Pi }\int_{-h/2}^{h/2}s_{\alpha \beta }{\bar{e}}_{\alpha \beta }dzda=\int_{\Pi }p{\bar{u}}_{3}(x,\frac{h}{2})da\;,$$

Which is satisfied for arbitrary virtual displacements (2), under the assumption that these displacements are neglected on the boundary and sufficiently regular and independent functions; moreover: *da* = *dx*_1_*dx*_2_.

The plate properties, i.e., stiffness tensor *d*_αβγδ_, inertia properties: μ, *j*, are periodic functions in **x**, given by:

(7) $$d_{\alpha \beta \gamma \delta }(x)=\int_{-h/2}^{h/2}c_{\alpha \beta \gamma \delta }(x,z)z^{2}dz,\;\;\;\;\;\mu (x)=\int_{-h/2}^{h/2}\rho (x,z)dz,\;\;\;\;\;j(x)=\int_{-h/2}^{h/2}\rho (x,z)z^{2}dz.$$

Combining the above assumptions (1)–(4) together with the virtual work Equation (6), after some manipulations using the divergence theorem and the du Bois-Reymond lemma to (6), *the governing equation of thin elastic periodic plates* takes the form:

(8) $${\left(d_{\alpha \beta \gamma \delta }w_{,\gamma \delta }\right)}_{,\alpha \beta }+\mu \ddot{w}-j{\ddot{w}}_{,\alpha \alpha }=p.$$

Periodic plates coefficients of Equation (8) are usually discontinuous and highly oscillating, periodic functions in **x**. It is very difficult to find solutions to this equation.

Hence, in this note, the original equation is replaced by a system of equations with constant coefficients, which can describe (or not) the information about the microstructure using the tolerance averaging method.

### 2.2. Foundations of the Tolerance Modelling

#### 2.2.1. Introductory Concepts

Some introductory concepts are applied in *the tolerance modelling method*. These concepts can be found in the book [24] and they are formulated for beams in [38] or for non-periodic plates in [49,53]. Hence, here they can be only mentioned: the averaging operator <·>, the tolerance parameter, the tolerance-periodic function *TP*(Π,Δ), the slowly-varying function *SV*(Π,Δ), the highly oscillating function *HO*(Π,Δ), and the fluctuation shape function *FS*(Π,Δ). Below, let us restate the averaging operator.

Let Δ(**x**) = **x** + Δ denote a cell at **x** ∈ Π_Δ_, Π_Δ_ = {**x** ∈ Π: Δ(**x**) ⊂ Π}. *The averaging operator*, one of the fundamental concepts of the tolerance method, is defined by

(9) $$<f>(x)={\left(l_{1}l_{2}\right)}^{-1}\int_{\Delta (x)}f(y_{1},y_{2})dy_{1}dy_{2},\;x\in \Pi_{\Delta },\;y\in \Delta (x),$$

for an integrable function *f*. For function *f*, being periodic in **x** its averaged value from (9) is constant.

A very important concept is *a fluctuation shape function*. It is a function, *g*(·), being continuous together with gradient *∂*^1^*g* and with a piecewise continuous and bounded gradient ∂^2^*g*; depending on *l* as a parameter, which satisfies proper conditions, i.e., g∈FS(Π,Δ) if conditions hold:

(10) $$\begin{array}{l} (i)\;\partial^{k}g\in O(l^{\alpha -k})\;\mathrm{for}\;k=0,1,\ldots ,\alpha ,\;\alpha =2,\;\partial^{0}g\equiv g,\\ (ii)\;<g>(x)\approx 0\;\forall x\in \Pi_{\Delta }, \end{array}$$

where *l* is the microstructure parameter. Condition (10*ii*) can be replaced by <μ*g*>(**x**) ≈ 0 for every **x** ∈ Π_Δ_, where μ > 0 is a certain tolerance-periodic function.

#### 2.2.2. Tolerance Modelling Assumptions

The fundamental tolerance modelling assumptions in their general form are formulated in the book [24]. For thin plates with a microstructure, they can be found, e.g., in [26,40,49]. Here, these assumptions are written below in the form for thin periodic plates.

One of these assumptions is *the micro-macro decomposition*, in which the deflection is assumed that can be decomposed in the form:

(11) $$w(x,t)=W(x,t)+g^{A}(x)V^{A}(x,t),\;A=1,\ldots ,N,$$

with the basic unknowns: *W*(·,*t*) called *the macrodeflection*, *V^A^*(·,*t*) called *the fluctuation amplitudes*, $W(\cdot ,t),\;V^{A}(\cdot ,t)\in SV(\Pi ,\Delta )$; and the known fluctuation shape functions $g^{A}(\cdot )\in FS(\Pi ,\Delta )$. Fluctuation shape functions can be derived as solutions to eigenvalue problems posed on the periodicity cell. However, in most cases they can be assumed in an approximate form as trigonometric functions [26,53] or saw-type functions [49].

Similar assumptions to (11) should be introduced for virtual displacements $\bar{w}(\cdot )$:

(12) $$\bar{w}(x)=\bar{W}(x)+g^{A}(x){\bar{V}}^{A}(x),\;A=1,\ldots ,N,$$

with slowly-varying functions $\bar{W}(\cdot ),\;{\bar{V}}^{A}(\cdot )\in SV(\Pi ,\Delta )$.

The second modelling assumption is *the tolerance averaging approximation*, such that terms *O*(δ) are negligibly small in the course of modelling, and they can be neglected in the following formulas:

(13) $$\begin{array}{l} \begin{array}{ll} \left(i\right) & <\phi >(x)=<\bar{\phi }>(x)+O(\delta ),\\ \left(ii\right) & <\phi F>(x)=<\phi >(x)F(x)+O(\delta ),\\ \left(iii\right) & <\phi (gF){,}_{\gamma }>(x)=<\phi g{,}_{\gamma }>(x)F(x)+O(\delta ), \end{array}\\ x\in \Pi ;\;\;\;\gamma =1,\alpha ;\;\alpha =1,2;\;0<\delta <<1;\;\phi \in TP(\Pi ,\Delta ),\;F\in SV(\Pi ,\Delta ),\;g\in FS(\Pi ,\Delta ). \end{array}$$

#### 2.2.3. Outline of the Modelling Procedure

In the modelling procedure, the above concepts and basic assumptions are applied (11)–(13). The procedure can be divided into a few steps.

The first step stands a substitution of micro-macro decompositions (11) and (12) into the virtual work Equation (6). In the next step the resulting equation is averaged using the averaging operation (9) over a periodicity cell, cf. [26]. In the third step, the tolerance averaged virtual work equation is obtained after using Formula (13) of *the tolerance averaging approximation*. Introducing the following denotations of averaged parameters, which can be stand averaged constitutive relations:

(14) $$M_{\alpha \beta }\equiv -<\int_{-h/2}^{h/2}s_{\alpha \beta }zdz>,\;M^{A}\equiv -<g_{,\alpha \beta }^{A}\int_{-h/2}^{h/2}s_{\alpha \beta }zdz>,$$

the tolerance averaged virtual work equation takes the form:

(15) $$\begin{array}{l} \int_{\Pi }(<\mu >\ddot{W}+<\mu g^{B}>{\ddot{V}}^{B})\delta Wda+\int_{\Pi }(<\mu g^{A}>\ddot{W}+<\mu g^{A}g^{B}>{\ddot{V}}^{B})\delta V^{A}da-\\ -\int_{\Pi }(<j>{\ddot{W}}_{,\alpha \alpha }+<jg_{,\alpha }^{B}>{\ddot{V}}_{,\alpha }^{B})\delta Wda+\int_{\Pi }(<jg_{,\alpha }^{A}>{\ddot{W}}_{,\alpha }+<jg_{,\alpha }^{A}g_{,\alpha }^{B}>{\ddot{V}}^{B})\delta V^{A}da+\\ +\int_{\Pi }M_{\alpha \beta ,\alpha \beta }\delta Wda+\int_{\Pi }M^{A}\delta V^{A}da=\int_{\Pi }p\delta Wda. \end{array}$$

At the end, after some manipulations governing equations of the tolerance model are obtained using the divergence theorem and the du Bois-Reymond lemma to Equation (15).

### 2.3. Governing Equations

#### 2.3.1. Tolerance Model Equations

Let us denote:

(16) $$\begin{array}{lll} D_{\alpha \beta \gamma \delta }\equiv <d_{\alpha \beta \gamma \delta }>, & D_{\alpha \beta }^{A}\equiv <d_{\alpha \beta \gamma \delta }g_{,\gamma \delta }^{A}>, & D^{AB}\equiv <d_{\alpha \beta \gamma \delta }g_{,\alpha \beta }^{A}g_{,\gamma \delta }^{B}>,\\ m\equiv <\mu >, & m^{A}\equiv l^{-2}<\mu g^{A}>, & m^{AB}\equiv l^{-4}<\mu g^{A}g^{B}>,\\ \vartheta \equiv <j>, & \vartheta_{\alpha }^{A}\equiv l^{-1}<jg_{,\alpha }^{A}>, & \vartheta_{\alpha \beta }^{AB}\equiv l^{-2}<jg_{,\alpha }^{A}g_{,\beta }^{B}>,\\ P\equiv <p>, & P^{A}\equiv l^{-2}<pg^{A}>. & \end{array}$$

The tolerance modelling procedure leads to the system of equations for the macrodeflection *W* and the fluctuation amplitudes of the deflection *V^A^*:

(17) $$\begin{array}{l} ({D_{\alpha \beta \gamma \delta }W_{,\gamma \delta }+D_{\alpha \beta }^{A}V^{A})}_{,\alpha \beta }+m\ddot{W}+l^{2}m^{A}{\ddot{V}}^{A}-\vartheta {\ddot{W}}_{,\alpha \alpha }-l\vartheta_{\alpha }^{A}{\ddot{V}}_{,\alpha }^{A}=P,\\ D_{\alpha \beta }^{A}W_{,\gamma \delta }+D^{AB}V^{B}+l^{2}m^{A}\ddot{W}+l\vartheta_{\alpha }^{A}{\ddot{W}}_{,\alpha }+l^{2}(l^{2}m^{AB}+\vartheta_{\alpha \beta }^{AB}){\ddot{V}}^{B}=l^{2}P^{A}. \end{array}$$

The above Equation (17) has constant coefficients and, together with micro-macro decompositions (11) and (12), constitutes *the tolerance model of thin elastic periodic plates*. This model allows us to describe the effect of the microstructure size on the plate dynamics by terms with the microstructure parameter *l*. It can be observed that the basic unknowns of Equation (17) have to be slowly varying functions in **x**, $W(\cdot ,t),\;V^{A}(\cdot ,t)\in SV(\Pi ,\Delta )$. Moreover, there have to be determined boundary conditions for the macrodeflection *W*.

#### 2.3.2. Asymptotic Model Equations

In order to compare results, the asymptotic model equations can be obtained. These equations can be derived using the formal asymptotic modelling procedure. However, below this is made by simple neglecting terms of an order of *O*(*l^n^*), *n* = 1,2,…, in Equation (17).

Hence, after these manipulations, Equation (17) is simplified to the form of *the asymptotic model* equations:

(18) $$\begin{array}{l} {\left(D_{\alpha \beta \gamma \delta }W_{,\gamma \delta }+D_{\alpha \beta }^{A}V^{A}\right)}_{,\alpha \beta }+m\ddot{W}-\vartheta {\ddot{W}}_{,\alpha \alpha }=P,\\ D_{\alpha \beta }^{A}W_{,\gamma \delta }+D^{AB}V^{B}=0, \end{array}$$

with all constant coefficients.

Equation (18), together with micro-macro decompositions (11) and (12), constitutes *the asymptotic model of thin elastic periodic plates*. Similar boundary conditions should be formulated as in the tolerance model, i.e., only for the macrodeflection *W*. Moreover, it can be observed that Equation (18)_2_ stand a system of linear algebraic equations for the fluctuation amplitudes *V^A^*, *A* = 1, …, *N*.

## 3. Analysis of Free Vibrations of Periodic Plate Strips with Various Boundary Conditions

### 3.1. Introduction to the Example

**Figure 2:**
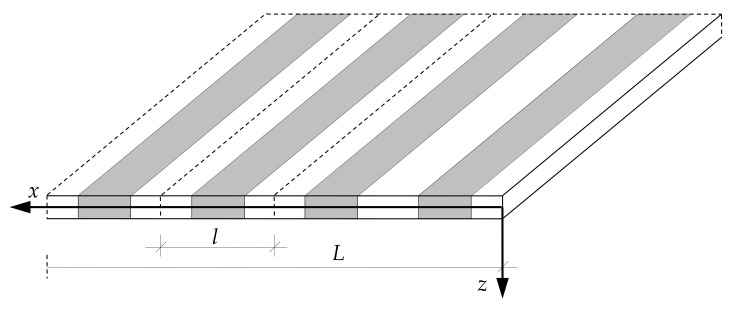
A fragment of a thin periodic plate strip.

Let us consider free vibrations of a thin periodic plate strip having span *L* along the *x*_1_-axis. The loading *p* is neglected, *p* = 0. The plate strip has periodic structure along its span, cf. Figure 2. However, all plate properties are independent of the *x*_2_-coordinate.

Hence, our considerations can be treated as independent of the *x*_2_-coordinate. Let us denote *x* = *x*_1_; *z* = *x*_3_; and ∂ ≡ (·),_1_; *x* ∈ [0,*L*]; *z* ∈ [−*h*/2,*h*/2], with a constant plate thickness *h*. The time derivatives are still denoted by overdots $\overset{\cdot }{\left(\cdot \right)}$. Let Δ≡[−l/2,l/2] be the basic cell in the interval ∧ ≡ [0, *L*], and *l*, *l* << *L*, be the length of this cell, satisfying the condition *l* << *L*. By Δ(x)≡[x−l/2,x+l/2] denote a cell with a centre at *x* ∈ [0, *L*].

It is assumed that the plate strip is made of two elastic isotropic materials, described by: Young’s moduli *E*′, *E*′′, Poisson’s ratios ν′, ν′′ and mass densities ρ′, ρ′′, respectively. These materials are perfectly bonded on interfaces and periodically distributed along the *x*-axis.

**Figure 3:**
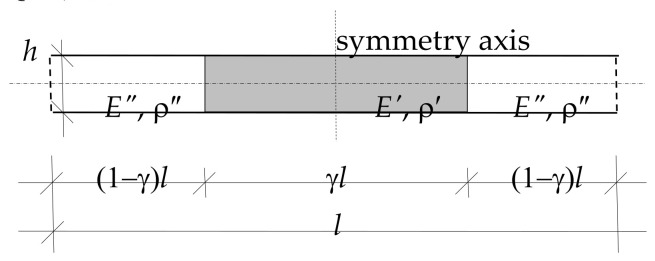
A periodicity cell of the plate strip under consideration.

It is assumed that the properties of the plate strip are described by the functions:

(19) $$E(y)=\left\{\begin{array}{lll} E^{\prime }, & \mathrm{for} & y\in ((1-\gamma )l/2,(1+\gamma )l/2),\\ E^{\prime \prime }, & \mathrm{for} & y\in [0,(1-\gamma )l/2]\cup [(1+\gamma )l/2,l], \end{array}\right.$$

(20) $$\rho (y)=\left\{\begin{array}{lll} \rho^{\prime }, & \mathrm{for} & y\in ((1-\gamma )l/2,(1+\gamma )l/2),\\ \rho^{\prime \prime }, & \mathrm{for} & y\in [0,(1-\gamma )l/2]\cup [(1+\gamma )l/2,l], \end{array}\right.$$

(21) $$\nu (y)=\left\{\begin{array}{lll} \nu^{\prime }, & \mathrm{for} & y\in ((1-\gamma )l/2,(1+\gamma )l/2),\\ \nu^{\prime \prime }, & \mathrm{for} & y\in [0,(1-\gamma )l/2]\cup [(1+\gamma )l/2,l], \end{array}\right.$$

with a distribution parameter of material properties γ, cf. Figure 3. Under the above assumptions, the effect of material periodic structure on free vibration frequencies will be considered with using ratios: *E*″/*E*′ ∈ [0, 1], ν″/ν′ ∈ [0, 1], ρ″/ρ′ ∈ [0, 1], *h*/*l* ∈ (0, 0.1], γ ∈ [0, 1].

Effects of the above parameters will be shown only for the first vibration—lower and higher (for the tolerance model). Hence, the considerations can be restricted to assume only one fluctuation shape function, i.e., *A* = *N* = 1. The analysis for these functions can be found in [24]. Denote *g ≡ g*^1^, *V ≡ V*^1^, *∂* ≡ (∙),_1_. Micro-macro decomposition (11) of the deflection *w*(*x*,*t*) of the plate strip is given by:

(22) w(x,t)=W(x,t)+g(x)V(x,t),

where W(⋅,t), V(⋅,t)∈SV(Λ,Δ) for every $t\in (t_{0},t_{1})$, g(⋅)∈FS(Λ,Δ).

Because the cell is assumed in the form shown in Figure 3, i.e., it has a symmetry axis normal to the midplane, the fluctuation shape function *g*(*x*) is assumed as

(23) $$g(y)=l^{2}[\cos(2\pi y/l)+c],\;y\in \Delta (x),\;x\in \Lambda ,$$

where the constant *c* is determined by the condition <μg>=0 and has the form:

(24) $$c=\frac{\sin(\pi \gamma )(\rho^{\prime }-\rho^{\prime \prime })}{\pi [\rho^{\prime }\gamma +\rho^{\prime \prime }(1-\gamma )]}$$

with γ as the constant distribution parameter of material properties.

Using definitions of periodic Function (7) for the considered plate strip, it can be written:

(25) $$d(x)\equiv \frac{h^{3}}{12[1-\nu {\left(x\right)}^{2}]}E(x),\mu (x)\equiv h\rho (x),j(x)\equiv \frac{h^{3}}{12}\rho (x).$$

It can be observed that for the assumed periodic cell, Figure 3, and the assumed fluctuation shape Function (23), term (16)_8_ neglects:

<j∂g>=0.

Denoting:

(26) $$\begin{array}{lll} \overset{⌣}{D}=<d>, & \overset{⌢}{D}=<d\partial \partial g>, & \bar{D}=<d\partial \partial g\partial \partial g>,\\ \overset{⌣}{\mu }=<\mu >, & \bar{\mu }=l^{-4}<\mu gg>, & \overset{⌣}{\vartheta }=<\vartheta >,\;\bar{\vartheta }=l^{-2}<\vartheta \partial g\partial g>, \end{array}$$

tolerance model Equation (17) has the form:

(27) $$\begin{array}{l} \partial \partial (\overset{⌣}{D}\partial \partial W+\overset{⌢}{D}V)+\overset{⌣}{\mu }\ddot{W}-\overset{⌣}{\vartheta }\partial \partial \ddot{W}=0,\\ \overset{⌢}{D}\partial \partial W+\bar{D}V+l^{2}(l^{2}\bar{\mu }+\bar{\vartheta })\ddot{V}=0. \end{array}$$

Equation (27) describe free vibrations of the periodic plate strip within the tolerance model.

Using denotations (26) for the plate strip under consideration, Equation (18)_1_ takes the form

(28) $$\partial \partial [(\overset{⌣}{D}-{\overset{⌢}{D}}^{2}/\bar{D})\partial \partial W]+\overset{⌣}{\mu }\ddot{W}-\overset{⌣}{\vartheta }\partial \partial \ddot{W}=0.$$

The above equation describes free vibrations of this plate strip in the framework of the asymptotic model.

### 3.2. The Using of the Ritz Method

In order to solve the problem of free vibrations and to find free vibration frequencies for different boundary conditions, the known Ritz method is applied, cf. [49,53]. In this method, formulas of the maximal strain energy $Y_{\max}$ and the maximal kinetic energy $K_{\max}$ should be determined.

The solutions to Equations (28) and (27) for the plate strip under consideration can be assumed as:

(29) $$W(x,t)=A_{W}\Psi (\alpha x)\cos(\omega t),V(x,t)=A_{V}\Theta (\alpha x)\cos(\omega t),$$

where α is a wave number, ω is a free vibration frequency, *A_W_* and *A_V_* are amplitudes. Functions Ψ(·) and Θ(·) are eigenvalue functions for the macrodeflection and the fluctuation amplitude, respectively, which have to satisfy the given boundary conditions for *x* = 0, *L*. To simplify, let us denote the first and second order derivatives of functions Ψ(·) and Θ(·) by:

(30) $$\partial \Psi (\alpha x)\equiv \alpha \tilde{\Psi }(\alpha x),\;\;\partial \Theta (\alpha x)\equiv \alpha \tilde{\Theta }(\alpha x),\;\;\partial \partial \Psi (\alpha x)\equiv \alpha^{2}\bar{\Psi }(\alpha x),\;\;\partial \partial \Theta (\alpha x)\equiv \alpha^{2}\bar{\Theta }(\alpha x).$$

In the following analysis four different cases of boundary conditions of the plate strip will be considered, i.e.:

- the simply supported plate strip
  (31) Ψ(0)=∂∂Ψ(0)=Ψ(L)=∂∂Ψ(L)=0;
- the clamped-hinged plate strip
  (32) Ψ(0)=∂Ψ(0)=Ψ(L)=∂∂Ψ(L)=0;
- the plate strip clamped on both edges
  (33) Ψ(0)=∂Ψ(0)=Ψ(L)=∂Ψ(L)=0;
- the cantilever plate strip
  (34) Ψ(0)=∂Ψ(0)=∂∂Ψ(L)=∂∂∂Ψ(L)=0.

The eigenvalue functions Ψ(·) and Θ(·) from solutions (29) can be assumed as for the homogeneous plate strip as functions satisfying the boundary conditions (31)–(34). In order to describe these solutions, the Krylov–Prager functions are introduced:

(35) $$\begin{array}{ll} S(\alpha x)=\frac{1}{2}[\cosh(\alpha x)+\cos(\alpha x)], & T(\alpha x)=\frac{1}{2}[\sinh(\alpha x)+\sin(\alpha x)],\\ W(\alpha x)=\frac{1}{2}[\cosh(\alpha x)-\cos(\alpha x)], & V(\alpha x)=\frac{1}{2}[\sinh(\alpha x)-\sin(\alpha x)]. \end{array}$$

Hence, the aforementioned eigenvalue functions Ψ(·) and Θ(·) can be written as:

- the simply supported plate strip
  (36) Ψ(αx)=sin(αx), Θ(αx)=sin(αx);
- the clamped-hinged plate strip
  (37) Ψ(αx)=Θ(αx)=W(αx)−coth(αL)V(αx);
- the plate strip clamped on both edges
  (38) $$\Psi (\alpha x)=\Theta (\alpha x)=W(\alpha x)-\frac{\cosh(\alpha L)-\cos(\alpha L)}{\sinh(\alpha L)-\sin(\alpha L)}V(\alpha x);$$
- the cantilever plate strip
  (39) $$\Psi (\alpha x)=\Theta (\alpha x)=W(\alpha x)-\frac{\sinh(\alpha L)-\sin(\alpha L)}{\cosh(\alpha L)+\cos(\alpha L)}V(\alpha x).$$

Now, applying the Ritz method formulas for the maximal strain energy $Y_{\max}$ and the maximal kinetic energy $K_{\max}$ should be formulated in the framework of both models—the tolerance and the asymptotic. Then, the conditions of the Ritz method have to be used:

(40) $$\frac{\partial (Y_{\max}-K_{\max})}{\partial A_{W}}=0,\;\frac{\partial (Y_{\max}-K_{\max})}{\partial A_{V}}=0,$$

from which formulas of free vibrations frequencies can be determined.

Denotations (26), after substituting into them the fluctuation shape function (23), functions of material properties (19)–(21) and eigenvalue functions Ψ(·) and Θ(·), take the form:


(41) $$\begin{array}{cc} \overset{⌣}{D}=\frac{h^{3}}{12}[\frac{E^{\prime \prime }}{1-{\nu^{\prime \prime }}^{2}}(1-\gamma )+\gamma \frac{E^{\prime }}{1-{\nu^{\prime }}^{2}}]\int_{0}^{L}{\left[\bar{\Psi }(\alpha x)\right]}^{2}dx, & \overset{⌢}{D}=\frac{\pi h^{3}}{3}(\frac{E^{\prime }}{1-{\nu^{\prime }}^{2}}-\frac{E^{\prime \prime }}{1-{\nu^{\prime \prime }}^{2}})\sin(\pi \gamma )\int_{0}^{L}\bar{\Psi }(\alpha x)\Theta (\alpha x)dx,\\ \bar{D}=\frac{{\left(\pi h\right)}^{3}}{3}\{(\frac{E^{\prime }}{1-{\nu^{\prime }}^{2}}-\frac{E^{\prime \prime }}{1-{\nu^{\prime \prime }}^{2}})[2\pi \gamma +\sin(2\pi \gamma )]+2\pi \frac{E^{\prime \prime }}{1-{\nu^{\prime \prime }}^{2}}\}\int_{0}^{L}{\left[\Theta (\alpha x)\right]}^{2}dx, & \\ \overset{⌣}{\mu }=h[(1-\gamma )\rho^{\prime \prime }+\gamma \rho^{\prime }]\int_{0}^{L}{\left[\Psi (\alpha x)\right]}^{2}dx, & \overset{⌣}{\vartheta }=\frac{h^{3}}{12}[(1-\gamma )\rho^{\prime \prime }+\gamma \rho^{\prime }]\int_{0}^{L}{\left[\tilde{\Psi }(\alpha x)\right]}^{2}dx,\\ \bar{\mu }=\frac{h}{4\pi }\{(\rho^{\prime }-\rho^{\prime \prime })[2\pi \gamma +\sin(2\pi \gamma )]+2\pi \rho^{\prime \prime }\}\int_{0}^{L}{\left[\Theta (\alpha x)\right]}^{2}dx+ & \\ \;\;+\frac{h}{\pi }(\rho^{\prime }-\rho^{\prime \prime })c[\pi c\gamma -2\sin(\pi \gamma )]\int_{0}^{L}{\left[\Theta (\alpha x)\right]}^{2}dx+h\rho^{\prime \prime }c^{2}\int_{0}^{L}{\left[\Theta (\alpha x)\right]}^{2}dx, & \\ \bar{\vartheta }=\frac{\pi h^{3}}{12}\{(\rho^{\prime }-\rho^{\prime \prime })[2\pi \gamma -\sin(2\pi \gamma )]+2\pi \rho^{\prime \prime }\}\int_{0}^{L}{\left[\Theta (\alpha x)\right]}^{2}dx. & \end{array}$$

The maximal strain energy $Y_{\max}$ and the maximal kinetic energy $K_{\max}$ by the tolerance model can be written as:

(42) $$Y_{\max}^{TM}=\frac{1}{2}[(\overset{⌣}{D}A_{W}{}^{2}\alpha^{2}+2\overset{⌢}{D}A_{W}A_{V})\alpha^{2}+\bar{D}A_{V}{}^{2}],\;K_{\max}^{TM}=\frac{1}{2}[A_{W}{}^{2}(\overset{⌣}{\mu }+\overset{⌣}{\vartheta }\alpha^{2})+A_{V}{}^{2}l^{2}(l^{2}\bar{\mu }+\bar{\vartheta })]\omega^{2}.$$

However, in the framework of the asymptotic model, these formulas have the form:

(43) $$Y_{\max}^{AM}=\frac{1}{2}[(\overset{⌣}{D}A_{W}{}^{2}\alpha^{2}+2\overset{⌢}{D}A_{W}A_{V})\alpha^{2}+\bar{D}A_{V}{}^{2}],\;K_{\max}^{AM}=\frac{1}{2}A_{W}{}^{2}(\overset{⌣}{\mu }+\overset{⌣}{\vartheta }\alpha^{2})\omega^{2}.$$

Using conditions (40) to relations (42), after some manipulations, the following formulas are obtained:

(44) $$\begin{array}{ll} {\left(\omega_{-,+}\right)}^{2} & \equiv \frac{l^{2}(l^{2}\bar{\mu }+\bar{\vartheta })\alpha^{4}\overset{⌣}{D}+(\overset{⌣}{\mu }+\alpha^{2}\overset{⌣}{\vartheta })\bar{D}}{2(\overset{⌣}{\mu }+\alpha^{2}\overset{⌣}{\vartheta })l^{2}(l^{2}\bar{\mu }+\bar{\vartheta })}\mp \\ & \mp \frac{\sqrt{{\left[l^{2}(l^{2}\bar{\mu }+\bar{\vartheta })\alpha^{4}\overset{⌣}{D}-(\overset{⌣}{\mu }+\alpha^{2}\overset{⌣}{\vartheta })\bar{D}\right]}^{2}+4{\left(\alpha^{2}\overset{⌢}{D}\right)}^{2}l^{2}(\overset{⌣}{\mu }+\alpha^{2}\overset{⌣}{\vartheta })(l^{2}\bar{\mu }+\bar{\vartheta })}}{2(\overset{⌣}{\mu }+\alpha^{2}\overset{⌣}{\vartheta })l^{2}(l^{2}\bar{\mu }+\bar{\vartheta })}, \end{array}$$

of *the lower* $\omega_{-}$ and *the higher* $\omega_{+}$ *free vibration frequencies*, respectively, in the framework of *the tolerance model*.

On the other side, the conditions (40) used to relations (43) of *the asymptotic model* led to the following formula:

(45) $$\omega^{2}=\alpha^{4}\frac{\overset{⌣}{D}\bar{D}-{\overset{⌢}{D}}^{2}}{\bar{D}(\overset{⌣}{\mu }+\overset{⌣}{\vartheta }\alpha^{2})}$$

of *the lower free vibration frequency* ω.

Under the above analytical results, it can be observed that in the framework of the tolerance model, the effect of the microstructure size of the plate strip can be analysed in the form of higher free vibration frequencies, (44)_2_, but the asymptotic model leads only to the fundamental lower frequency, (45).

### 3.3. Results of Calculations

Let us define dimensionless frequency parameters:

(46) $$\Omega_{-}{}^{2}\equiv \frac{\rho^{\prime }}{E^{\prime }}L^{2}\omega_{-}{}^{2},\;\Omega_{+}{}^{2}\equiv \frac{\rho^{\prime }}{E^{\prime }}L^{2}\omega_{+}{}^{2},\;\Omega^{2}\equiv \frac{\rho^{\prime }}{E^{\prime }}L^{2}\omega^{2},$$

where the free vibration frequencies ω_−_, ω_+_, and ω are determined by Equations (44) and (45), respectively.

Results of calculations are presented in Figure 4, Figure 5, Figure 6, Figure 7, Figure 8, Figure 9, Figure 10, Figure 11, Figure 12, Figure 13, Figure 14, Figure 15, Figure 16, Figure 17, Figure 18 and Figure 19.

**Figure 4:**
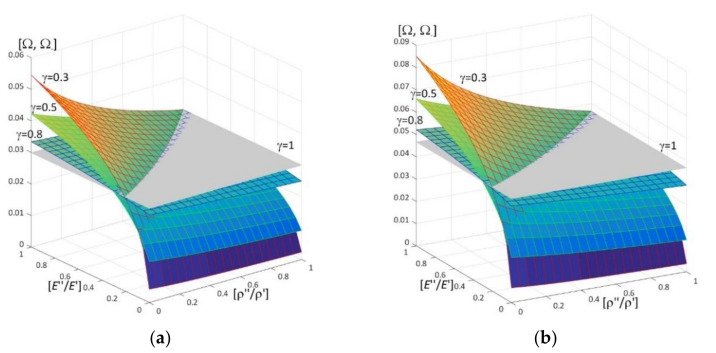
Dimensionless lower frequency parameters (46)_1,3_ versus pairs of ratios (ρ″/ρ′; *E*″/*E*′) (for *h*/*l* = 0.1, *l*/*L* = 0.1) for: (**a**) a simply supported plate strip, (**b**) a clamped-hinged plate strip.

**Figure 5:**
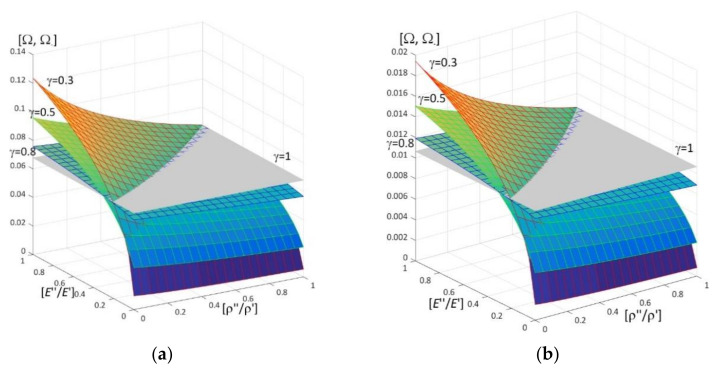
Dimensionless lower frequency parameters (46)_1,3_ versus pairs of ratios (ρ″/ρ′; *E*″/*E*′) (for *h*/*l* = 0.1, *l*/*L* = 0.1) for: (**a**) a clamped-clamped plate strip, (**b**) a cantilever plate strip.

**Figure 6:**
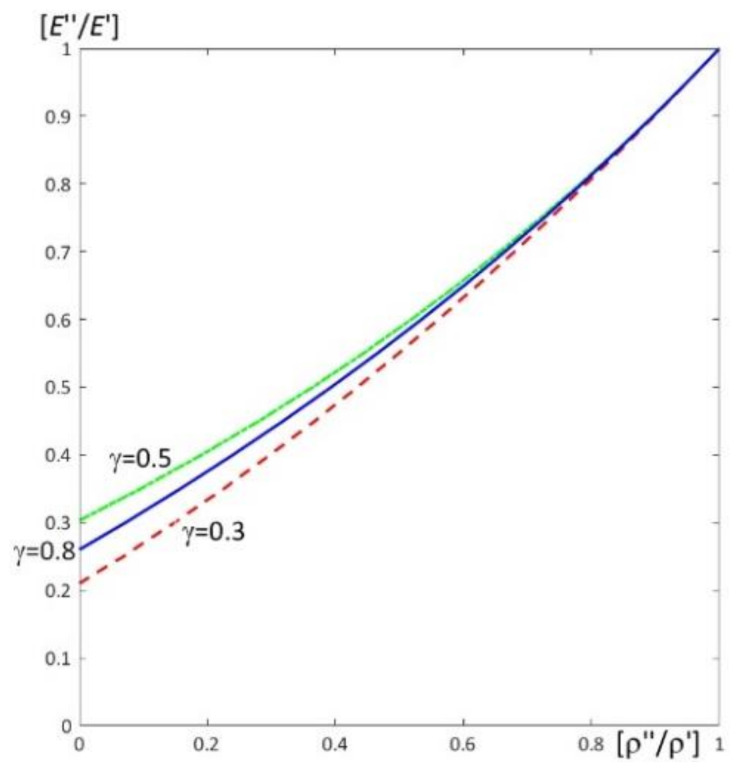
Traces of surfaces of dimensionless lower frequency parameters (46)_1,3_ from Figure 4 and Figure 5 on planes of these frequency parameters for the homogeneous plate strips (γ = 1) (for *h*/*l* = 0.1, *l*/*L* = 0.1) for all boundary conditions under consideration.

**Figure 7:**
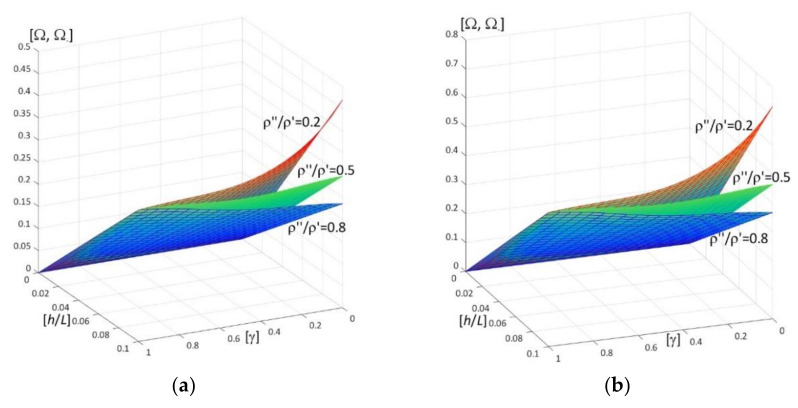
Dimensionless lower frequency parameters (46)_1,3_ versus pairs of ratios (γ; *h*/*L*) (for *E*″/*E*′ = 0.5, *l*/*L* = 0.1) for: (**a**) a simply supported plate strip, (**b**) a clamped-hinged plate strip.

**Figure 8:**
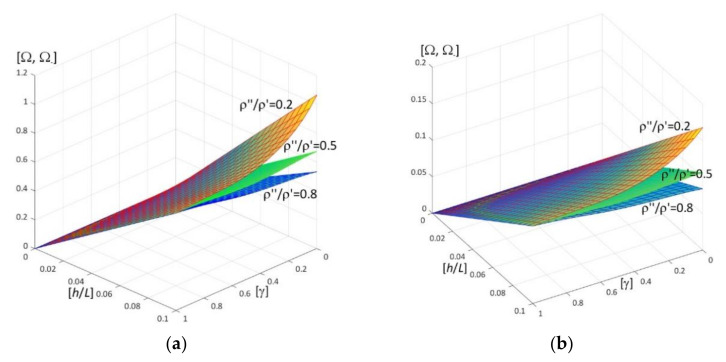
Dimensionless lower frequency parameters (46)_1,3_ versus pairs of ratios (γ; *h*/*L*) (for *E*″/*E*′ = 0.5, *l*/*L* = 0.1) for: (**a**) a clamped-clamped plate strip, (**b**) a cantilever plate strip.

**Figure 9:**
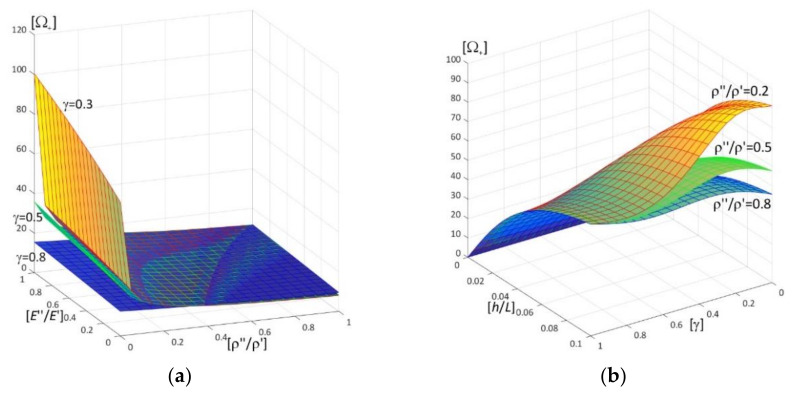
Dimensionless higher frequency parameters (46)_2_ versus: (**a**) pairs of ratios (ρ″/ρ′;*E*″/*E*′) (for *h*/*l* = 0.1, *l*/*L* = 0.1), (**b**) pairs of ratios (γ; *h*/*L*) (for *E*″/*E*′ = 0.5, *l*/*L* = 0.1).

**Figure 10:**
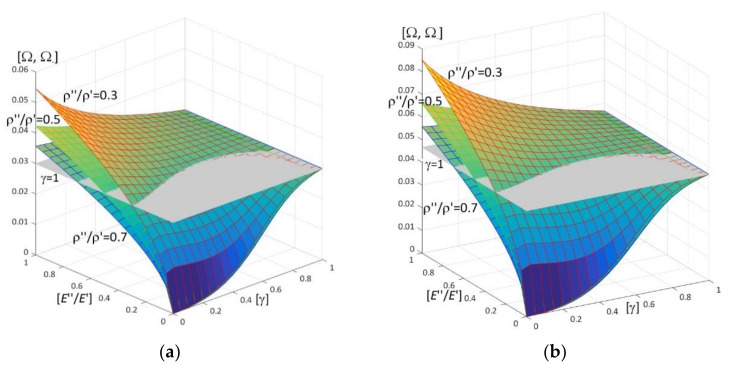
Dimensionless lower frequency parameters (46)_1,3_ versus pairs of ratios (γ; *E*″/*E*′) (for ν″/ν′ = 1, *l*/*L* = 0.1, *h*/*l* = 0.1) for: (**a**) a simply supported plate strip, (**b**) a clamped-hinged plate strip.

**Figure 11:**
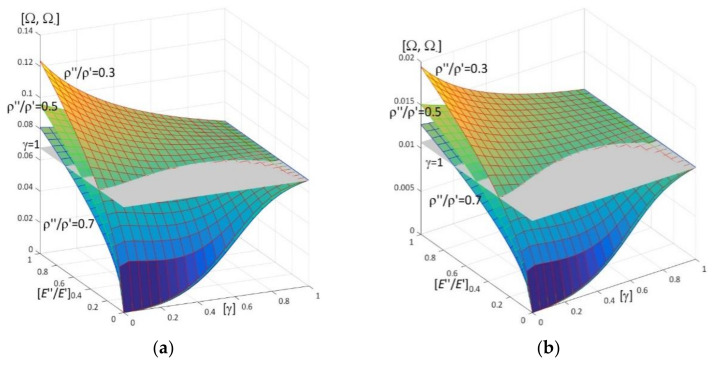
Dimensionless lower frequency parameters (46)_1,3_ versus pairs of ratios (γ; *E*″/*E*′) (for ν″/ν′ = 1, *l*/*L* = 0.1, *h*/*l* = 0.1) for: (**a**) a clamped-clamped plate strip, (**b**) a cantilever plate strip.

**Figure 12:**
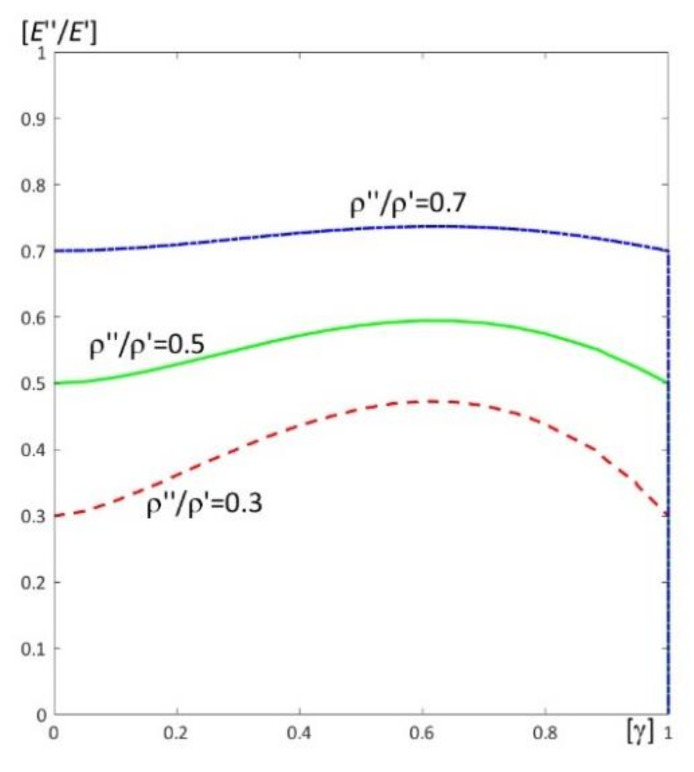
Traces of surfaces of dimensionless lower frequency parameters (46)_1,3_ from Figure 10 and Figure 11 on planes of these frequency parameters for the homogeneous plate strips (γ = 1) (for ν″/ν′ = 1, *h*/*l* = 0.1, *l*/*L* = 0.1) for all boundary conditions under consideration.

**Figure 13:**
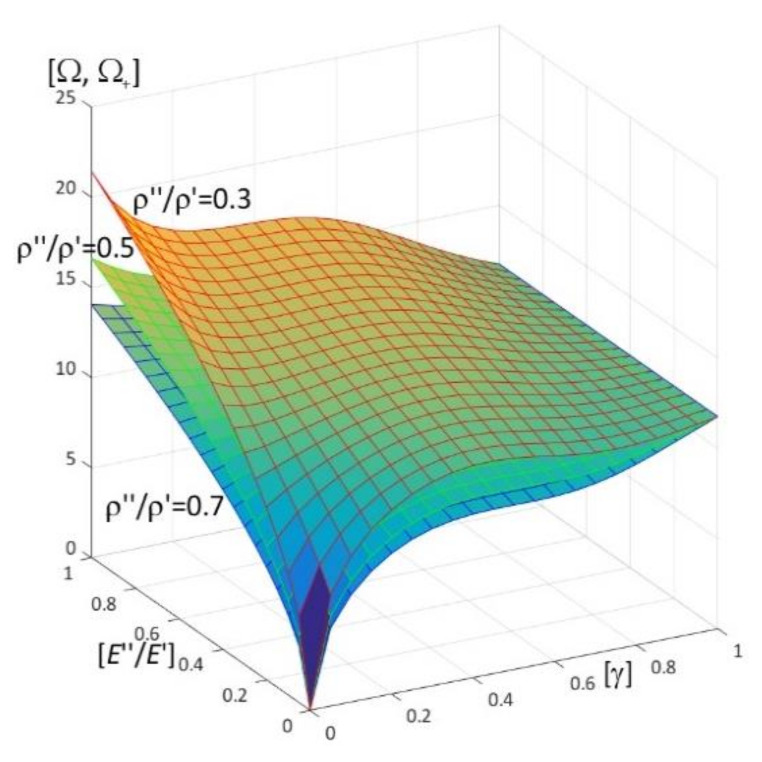
Dimensionless higher frequency parameters (46)_2_ versus pairs of ratios (γ; *E*″/*E*′) (for ν″/ν′ = 1, *l*/*L* = 0.1, *h*/*l* = 0.1).

**Figure 14:**
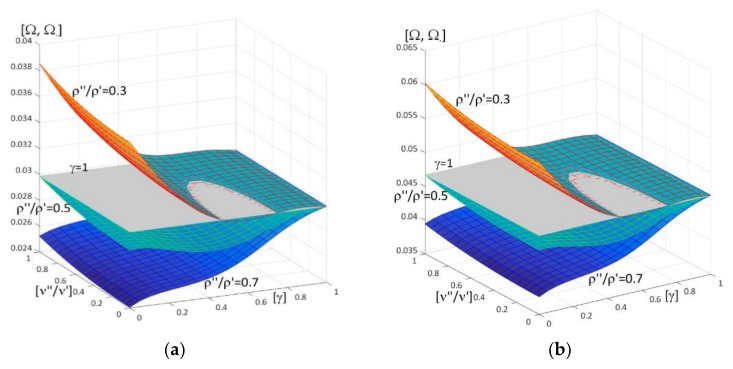
Dimensionless lower frequency parameters (46)_1,3_ versus pairs of ratios (γ; ν″/ν′) (for *E*″/*E*′ = 0.5, *l*/*L* = 0.1, *h*/*l* = 0.1) for: (**a**) a simply supported plate strip, (**b**) a clamped-hinged plate strip.

**Figure 15:**
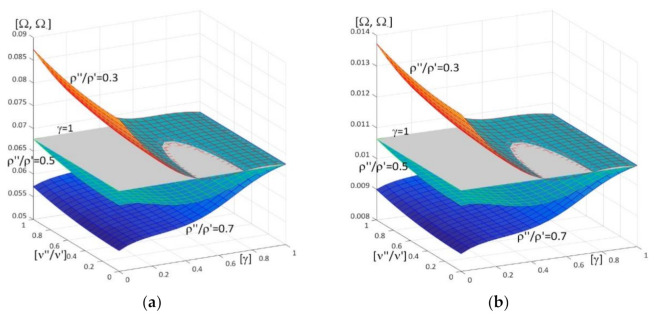
Dimensionless lower frequency parameters (46)_1,3_ versus pairs of ratios (γ; ν″/ν′) (for *E*″/*E*′ = 0.5, *l*/*L* = 0.1, *h*/*l* = 0.1) for: (**a**) a clamped-clamped plate strip, (**b**) a cantilever plate strip.

**Figure 16:**
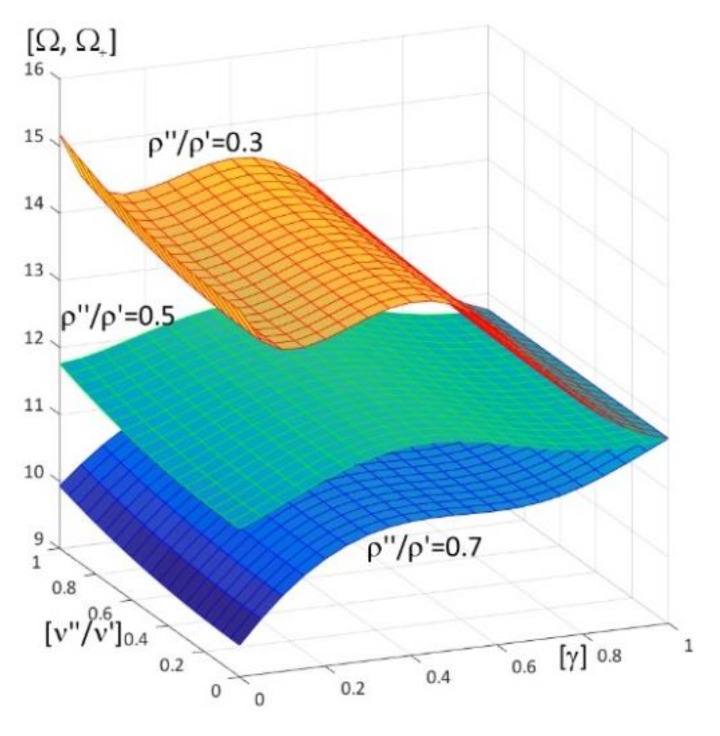
Dimensionless higher frequency parameters (46)_2_ versus pairs of ratios (γ; ν″/ν′) (for *E*″/*E*′ = 0.5, *l*/*L* = 0.1, *h*/*l* = 0.1).

**Figure 17:**
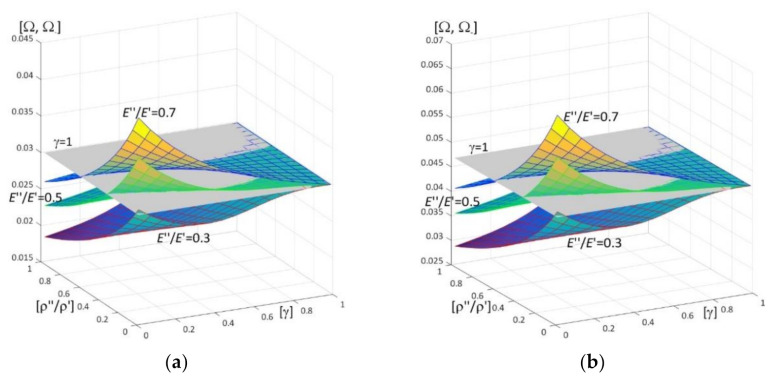
Dimensionless lower frequency parameters (46)_1,3_ versus pairs of ratios (γ; ρ″/ρ′) (for ν″/ν′ = 1, *l*/*L* = 0.1, *h*/*l* = 0.1) for: (**a**) a simply supported plate strip, (**b**) a clamped-hinged plate strip.

**Figure 18:**
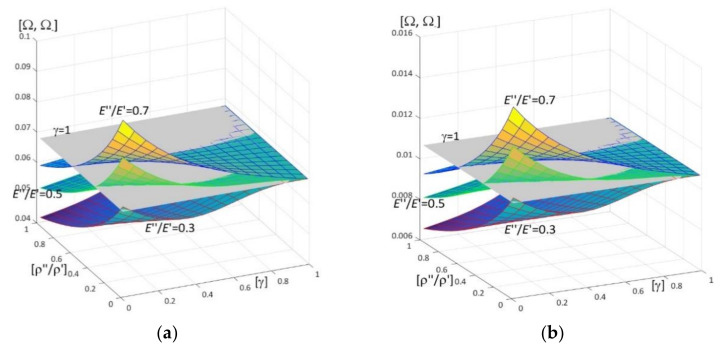
Dimensionless lower frequency parameters (46)_1,3_ versus pairs of ratios (γ; ρ″/ρ′) (for ν″/ν′ = 1, *l*/*L* = 0.1, *h*/*l* = 0.1) for: (**a**) a clamped-clamped plate strip, (**b**) a cantilever plate strip.

**Figure 19:**
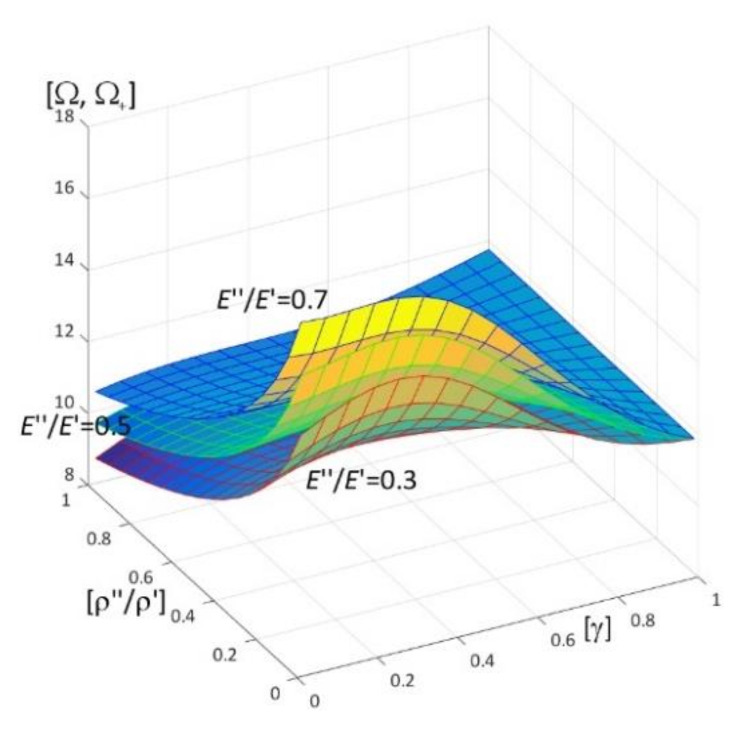
Dimensionless higher frequency parameters (46)_2_ versus pairs of ratios (γ; ρ″/ρ′) (for ν″/ν′ = 1, *l*/*L* = 0.1, *h*/*l* = 0.1).

Plots in Figure 4a, Figure 7a, Figure 10a, Figure 14a and Figure 17a are made for the simply supported plate strip, in Figure 4b, Figure 7b, Figure 10b, Figure 14b and Figure 17b for the clamped-hinged plate strip, in Figure 5a, Figure 8a, Figure 11a, Figure 15a and Figure 18a for the plate strip clamped on both edges, and in Figure 5b, Figure 8b, Figure 11b, Figure 15b and Figure 18b for the cantilever plate strip. Figure 4, Figure 5, Figure 6, Figure 7 and Figure 8, Figure 10, Figure 11 and Figure 12, Figure 14, Figure 15, Figure 17 and Figure 18 show results of the lower frequency parameters (46)_1,3_, but Figure 9, Figure 13, Figure 16 and Figure 19 are the higher frequency parameters (46)_2_.

The results shown in Figure 4 and Figure 5 and in Figure 9a are in the form of surfaces of the frequency parameters versus pairs of ratios (ρ″/ρ′; *E*″/*E*′). In calculations there are assumed: the Poisson’s ratios ν′′ = ν′ = 0.3, ratio *l*/*L* = 0.1, ratio *h*/*l* = 0.1 and γ = 0.3, 0.5, 0.8.

The lower frequency parameters of the homogeneous plate strips are presented in Figure 4, Figure 5, Figure 10, Figure 11, Figure 14, Figure 15, Figure 17 and Figure 18 by grey planes (γ = 1) and they are related to values Ω: 0.0298656 for the simply supported plate strip (Figure 4a, Figure 10a, Figure 14a and Figure 17a), 0.0466553 for the clamped-hinged plate strip (Figure 4b, Figure 10b, Figure 14b and Figure 17b), 0.0676988 for the clamped-clamped plate strip (Figure 5a, Figure 11a, Figure 15a and Figure 18a), and 0.0106397 for the cantilever plate strip (Figure 5b, Figure 11b, Figure 15b and Figure 18b).

The effect of differences of Young’s moduli (ratios *E*″/*E*′) and mass densities (ratios ρ″/ρ′) on the lower frequency parameters can be observed in Figure 4 and Figure 5, i.e., they increase with the increasing of ratio *E*″/*E*′; they decrease with the increasing of ratio ρ″/ρ′.

There are pairs of ratios ((ρ″/ρ′)*; (*E*″/*E*′)*) such that, cf. Figure 4 and Figure 5: the lower frequency parameters are smaller than this parameter for the homogeneous plate strip (γ = 1) for *E*″/*E*′ ≤ (*E*″/*E*′)* and ρ″/ρ′ ≥ (ρ″/ρ′)*; they are bigger than this parameter for the homogeneous plate strip (γ = 1) for *E*″/*E*′ > (*E*″/*E*′)* and ρ″/ρ′ < (ρ″/ρ′)*.

Figure 6 shows traces of surfaces of the lower frequency parameters (46)_1,3_, from Figure 4 and Figure 5 (pairs of ratios ((ρ″/ρ′)*; (*E*″/*E*′)*) as curves), on planes of these frequency parameters for the homogeneous plate strips (γ = 1) (for ν″/ν′ = 1, *h*/*l* = 0.1, *l*/*L* = 0.1) for all considered boundary conditions, mentioned above. It can be observed that these traces are the same for various boundary conditions of periodic plate strips under consideration, for fixed values of the parameter γ = 0.3, 0.5, 0.8.

However, Figure 7, Figure 8 and Figure 9b present surfaces of dimensionless frequency parameters versus pairs of ratios (γ; *h*/*L*), γ ∈ [0, 1], *h*/*l* ∈ (0, 0.1]. These calculations are made for: the Poisson’s ratios ν″ = ν′ = 0.3, ratio *E*″/*E*′ = 0.5, ratios ρ″/ρ′ = 0.2, 0.5, 0.8.

From Figure 7 and Figure 8, it can be observed that the lower frequency parameters increase with the increasing of *h*/*L* and decrease with the increasing of γ for certain values of the ratio ρ″/ρ′, e.g., ρ″/ρ′ = 0.2, but for other values of this ratio they are more varied (it is the parameter γ_0_ that the frequency parameters decrease for γ ≤ γ_0_ but increase for γ > γ_0_). All surfaces of the lower frequency parameters have the same edge for γ = 1.

Similar effects of the ratios ρ″/ρ′, *E*″/*E*′ as for the lower frequency parameters presented in Figure 4 and Figure 5 can also be found for the higher frequency parameters in Figure 9a, i.e., they increase with the increasing of ratio *E*″/*E*′; they decrease with the increasing of ratio ρ″/ρ′.

The dependency of the higher frequency parameters on the distribution parameter of material properties γ and the ratio of the thickness *h*/*L*, can be found in Figure 9b (for fixed ratios: *E*″/*E*′ = 0.5; ρ″/ρ′ = 0.2, 0.5, 0.8). It can be observed that the higher frequency parameters increase with the increasing of *h*/*L*; the dependency of these parameters on the parameter γ is more complicated, since for fixed ratios *E*″/*E*′ = 0.5 and ρ″/ρ′ = 0.2, 0.5, 0.8 there are γ_1_ and γ_2_ such that these frequency parameters increase for γ ≤ γ_1_ or γ > γ_2_, but decrease for γ_1_ < γ ≤ γ_2_. Similarly to lower frequency parameters, cf. Figure 7 and Figure 8, all surfaces of the higher frequency parameters have the same edge for γ = 1.

Surfaces of dimensionless frequency parameters versus pairs of ratios (γ; *E*″/*E*′), γ ∈ [0, 1], *E*″/*E*′ ∈ [0, 1], are shown in Figure 10, Figure 11 and Figure 13. These calculations are made for: the Poisson’s ratios ν″ = ν′ = 0.3, ratio *h*/*l* = 0.1, ratios ρ″/ρ′ = 0.3, 0.5, 0.7.

From surfaces of the lower frequency parameters versus pairs (γ; *E*″/*E*′) presented in Figure 10 and Figure 11, it can be observed that the frequency parameters increase with the increasing of *E*″/*E*′ and with the increasing of γ. Moreover, all surfaces of the lower frequency parameters have the same edge for γ = 1.

It can be distinguished that for pairs of ratios (γ*; (*E*″/*E*′)*) such as cf. Figure 10 and Figure 11, the lower frequency parameters are smaller than this parameter for the homogeneous plate strip (γ = 1) for some values γ* and *E*″/*E*′ ≤ (*E*″/*E*′)*(γ*), where (*E*″/*E*′)* is dependent on γ*; they are bigger than this parameter for the homogeneous plate strip (γ = 1) for *E*″/*E*′ > (*E*″/*E*′)*(γ*), where (*E*″/*E*′)* is dependent on γ*.

In Figure 12, traces of surfaces of the lower frequency parameters are presented (46)_1,3_, from Figure 10 and Figure 11, on planes related to the homogeneous plate strips (γ = 1) (for ν″/ν′ = 1, *h*/*l* = 0.1, *l*/*L* = 0.1) for all considered boundary conditions (the pairs of ratios (γ*; (*E*″/*E*′)*) from Figure 10 and Figure 11). Similarly to Figure 6, these traces are the same for various boundary conditions of these periodic plate strips, for fixed values of the ratio ρ″/ρ′ = 0.3, 0.5, 0.7.

The higher frequency parameters depend on the distribution parameter of material properties γ and the ratio *E*″/*E*′, cf. Figure 13 (for fixed ratios: ν″/ν′ = 1; ρ″/ρ′ = 0.3, 0.5, 0.7). From this figure it can be observed that the frequency parameters increase with the increasing of *E*″/*E*′. However, the dependency of these parameters on the parameter γ is more complicated, because for the fixed ratios ν″/ν′ = 1 and ρ″/ρ′ = 0.3, 0.5, 0.7, there are γ_1_ and γ_2_ such that these frequency parameters increase for γ ≤ γ_1_ or γ > γ_2_ but decrease for γ_1_ < γ ≤ γ_2_. Similarly to the lower frequency parameters, all surfaces of the higher frequency parameters have the same edge for γ = 1.

Surfaces of dimensionless frequency parameters versus pairs of ratios (γ; ν″/ν′), γ ∈ [0, 1], ν″/ν′ ∈ [0, 1], are presented in Figure 14 and Figure 15 (for lower frequencies) and in Figure 16 (for higher frequencies). These plots are made for ratios: *E*″/*E*′ = 0.5, *h*/*l* = 0.1, ρ″/ρ′ = 0.3, 0.5, 0.7.

From surfaces of the lower frequency parameters versus pairs (γ; ν″/ν′) presented in Figure 14 and Figure 15, it can be observed that:

- the frequency parameters increase with the increasing of ν″/ν′;
- the dependency of these parameters on the parameter γ is more complicated, since (for the fixed ratio *E*″/*E*′ = 0.5):
  - for some fixed values of ρ″/ρ′, e.g., ρ″/ρ′ = 0.7, these frequency parameters increase with the increasing of γ,
  - for some fixed values of ρ″/ρ′, e.g., ρ″/ρ′ = 0.5, there is γ_0_ such that these frequency parameters decrease for γ ≤ γ_0_, but increase for γ_0_ < γ,
  - for some fixed values of ρ″/ρ′, e.g., ρ″/ρ′ = 0.3, there are γ_1_ and γ_2_ such that these frequency parameters decrease for γ ≤ γ_1_ or γ > γ_2_, but increase for γ_1_ < γ ≤ γ_2_;
- values presented in surfaces of the lower frequency parameters are smaller than this parameter for the homogeneous plate strip (γ = 1) for—the fixed ratio *E*″/*E*′ = 0.5 and some fixed ratios ρ″/ρ′, ρ″/ρ′ > 0.3;
- for some fixed ratios ρ″/ρ′, ρ″/ρ′ ≤ 0.3, there are some values γ* and ν″/ν′ ≥ (ν″/ν′)*(γ*), where (ν″/ν′)* is dependent on γ*, such that the lower frequency parameters are bigger than this parameter for the homogeneous plate strip (γ = 1).
- Moreover, all surfaces of the lower frequency parameters have the same edge for γ = 1.

Figure 16 shows that the higher frequency parameters increase with the increasing of ν″/ν′. Moreover, the dependency of these parameters on the parameter γ is more complicated, since (for the fixed ratio *E*″/*E*′ = 0.5):

- for some fixed ratios ρ″/ρ′ = 0.5, 0.7 there are γ_1_ and γ_2_ such that these frequency parameters increase for γ ≤ γ_1_ or γ > γ_2_, but decrease for γ_1_ < γ ≤ γ_2_,
- however, this dependency of these parameters on the parameter γ for the rather small fixed ratio ρ″/ρ′, e.g., ρ″/ρ′ = 0.3, has other form, because there are γ_3_ and γ_4_ such that these frequency parameters decrease for γ ≤ γ_3_ or γ > γ_4_, but increase for γ_3_ < γ ≤ γ_4_.

Similarly to the lower frequency parameters, cf. Figure 14 and Figure 15, all surfaces of the higher frequency parameters have the same edge for γ = 1.

Similar graphs, but versus pairs of ratios (γ; ρ″/ρ′), γ ∈ [0, 1], ρ″/ρ′ ∈ [0, 1], can be found in Figure 17 and Figure 18 (for lower frequencies) and in Figure 19 (for higher frequencies). These results are obtained for parameters: ν″/ν′ = 1, ρ″/ρ′ = 0.5, *h*/*l* = 0.1, *E*″/*E*′ = 0.3, 0.5, 0.7.

From Figure 17 and Figure 18, it can be concluded that:

- the frequency parameters decrease with the increasing of ρ″/ρ′;
- the dependency of these parameters on the parameter γ is more complicated, because (for the fixed ratio ν″/ν′ = 1):
  - for some fixed ratios *E*″/*E*′, e.g., *E*″/*E*′ = 0.5, 0.7, the frequency parameters decrease with the increasing of the parameter γ,
  for some fixed ratios *E*″/*E*′, *E*″/*E*′ < 0.5, it is γ_0_ such that these frequency parameters decrease for γ ≤ γ_0_, but increase for γ_0_ < γ;
- the lower frequency parameters are smaller than this parameter for the homogeneous plate strip (γ = 1) for some values γ* and ρ″/ρ′ ≥ (ρ″/ρ′)*(γ*), where (ρ″/ρ′)* is dependent on γ*;
- the frequency parameters are bigger than this parameter for the homogeneous plate strip (γ = 1) for some γ* and ρ″/ρ′ < (ρ″/ρ′)*(γ*), where (ρ″/ρ′)^*^ depends on γ*.

Moreover, all surfaces of the lower frequency parameters have the same edge for γ = 1.

Figure 19 presents the higher frequency parameters increase with the decreasing of ρ″/ρ′. On the other hand, the dependency of these parameters on the parameter γ is more complicated, since (for the fixed ratio ν″/ν′ = 1):

- for more fixed ratios *E*″/*E*′ < 0.7 (e.g., *E*″/*E*′ = 0.3, 0.5) it is γ_0_ (dependent on ρ″/ρ′) such that these frequency parameters increase for γ ≤ γ_0_, but decrease for γ_0_ < γ,
- however, this dependency of these parameters on the parameter γ for the rather big fixed ratio *E*″/*E*′, e.g., *E*″/*E*′ = 0.7, has other form, because there are γ_1_ and γ_2_ such that these frequency parameters decrease for γ ≤ γ_1_ or γ > γ_2_, but increase for γ_1_ < γ ≤ γ_2_.

Similarly to the lower frequency parameters, cf. Figure 17 and Figure 18, all surfaces of the higher frequency parameters have the same edge for γ = 1.

Moreover, all calculations are made for the wave number α related to the first form of eigenfunction for every case of the supports, i.e.,: α = π for (31), Figure 4a, Figure 7a, Figure 10a, Figure 14a and Figure 17a; α = 3.9266 for (32), Figure 4b, Figure 7b, Figure 10b, Figure 14b and Figure 17b; α = 4.7300 for (33), Figure 5a, Figure 8a, Figure 11a, Figure 15a and Figure 18a; and α = 1.8751 for (34), Figure 5b, Figure 8b, Figure 11b, Figure 15b and Figure 18b.

It can be observed that the tolerance and the asymptotic models lead to nearly identical values of lower frequency parameters, cf. Figure 4, Figure 5, Figure 6, Figure 7, Figure 8, Figure 10, Figure 11, Figure 12, Figure 14, Figure 15, Figure 17 and Figure 18. Moreover, plots of surfaces of higher frequency parameters are the same for various boundary conditions of periodic plate strips under consideration.

## 4. Some Final Remarks

Vibrations of thin periodic plates for various boundary conditions have been modelled here. Using *the tolerance modelling*, the known differential equation of Kirchhoff-type plates has been averaged. This method leads from the governing differential equation with non-continuous, periodic coefficients to the system of differential equations with constant coefficients. Using this method, the effect of the microstructure size on the overall dynamic behaviour of the plates under consideration is taken into account in the derived tolerance model equations. On the other hand, a simplified averaged model—the asymptotic model—is described by the governing equation without this effect.

Under the presented considerations, the following general remarks, common to the application of the tolerance modelling method for different microheterogeneous objects, can be formulated.

1. *The tolerance model* allows us to analyse *the effect of the microstructure size* on dynamic problems of thin periodic plates under consideration, e.g., the “higher order” vibrations, related to the plate microstructure.
2. A certain *a posteriori* criterion of physical reliability for the model is that the basic unknowns *W*, *V^A^*, *A* = 1, …, *N*, have to be slowly-varying functions. Moreover, under these conditions, the governing equations of *the tolerance model* have a physical sense.
3. Using *the asymptotic model* of periodic plates, lower order (fundamental) vibrations can be only analysed.

Summarising the remarks of the results of calculations from Section 3, some additional, more general comments can be formulated:

1. Lower free vibration frequencies (also called fundamental frequencies) can be analysed within both the models—the tolerance and the asymptotic.
2. Values of lower free vibration frequencies of the considered periodic plate strips also depend on boundary conditions of these strips and they change from the highest values to the smallest as for the homogeneous plate strips with identical boundary conditions: the clamped-clamped plate strip, the clamped-hinged plate strip, the simply supported plate strip, the cantilever plate strip.
3. Higher free vibration frequencies, related to the periodic microstructure of the plate, can be investigated only within the tolerance model.
4. Values of higher free vibration frequencies of the considered periodic plate strips do not depend on boundary conditions of these plates, but only on material and geometrical properties of the plates.
5. The effects of differences between material or geometrical parameters, such that Young’s moduli (the ratio *E*″/*E*′), mass densities (the ratio ρ″/ρ′), Poisson’s ratios (the ratio ν″/ν′), the plate thickness (the ratio *h*/*L*), on free vibration frequencies are similar for both kinds of them—lower and higher.
6. The effect of the distribution parameter of material properties γ on these frequencies is more complicated to describe and is slightly different for both kinds of frequencies—lower and higher.
7. The effect of the parameter γ on the frequencies is related to the material properties Young’s moduli (the ratio *E*″/*E*′), mass densities (the ratio ρ″/ρ′), and Poisson’s ratios (the ratio ν″/ν′).
8. Sections by the planes corresponding to the fundamental free vibration frequency of homogeneous plate strips of surfaces for the lower frequencies of periodic plate strips are identical for all boundary conditions. Therefore, created in this way, traces of these surfaces of lower frequencies on the relevant planes of frequencies are the same for all considered boundary conditions.

In this paper, an application of the tolerance model to the analysis free vibration frequencies—lower and higher—are shown for periodic plate strips with various boundary conditions. Selected results were also compared with the results obtained by the finite element method, cf. Appendix A.

We presented a wide analysis of issues of free vibrations of periodic plates, which allows us to notice that the tolerance model seems to be a good tool in the study of this kind of problem. Moreover, this model can be used in the consideration of optimisation of periodic plates.

## Figures and Tables

**Figure A1:**
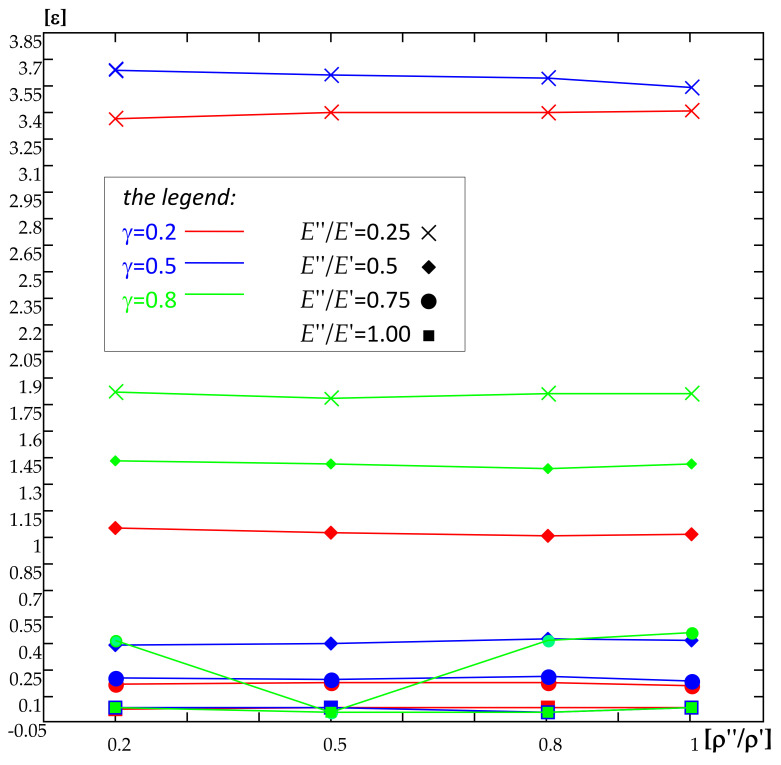
The parameter of differences ε (A1) versus the ratios ρ″/ρ′ (for ν″/ν′ = 1, *l*/*L* = 0.1, *h*/*l* = 0.1; ν″ = ν′ = 0.3; *E*″/*E*′ = 0.25, 0.5, 0.75; γ = 0.2, 0.5, 0.8).

## Appendix A

Here, obtained results for some selected cases of the simply supported plate strips are compared with results calculated using a commercial computer program of the finite element method (FEM)—Abaqus, with a type of elements S8R5. These results are shown in Table A1. The comparisons are made only for the fundamental lower frequency parameters, because they can only be calculated in the framework of the commercial computer programs of FEM. The parameter of differences between the results of the FEM (Ω_0_) and the tolerance model (Ω) is denoted by:

(A1) $$\varepsilon =|\frac{\Omega_{0}-\Omega_{\_}}{\Omega_{\_}}|100\%.$$

Results are obtained for these periodic plate strips for ratios or parameters: Poisson’s ratios ν″ = ν′ = 0.3; *E*″/*E*′ = 0.25, 0.5, 0.75; ρ″/ρ′ = 0.2, 0.5, 0.8; γ = 0.2, 0.5, 0.8. Additionally, the first lower frequency parameter for the proper homogeneous plate strip is shown with these boundary conditions (ν″ = ν′ = 0.3; *E*″/*E*′ = 1; ρ″/ρ′ = 1; γ = 1) calculated using the tolerance model (Ω = 0.02987), the classical analytical solution (Ω_C_ = 0.02987), and FEM (Ω_0_ = 0.02986) [%].

**Table A1:** Frequency parameters Ω_−_, Ω_0_ for lower free vibration frequencies for the simply supported plate strip (α = π, ν = 0.3, *l*/*L* = 0.1, *h*/*l* = 0.1).

γ		*E*’’/*E*’ = 0.25			*E*’’/*E*’ = 0.5			*E*’’/*E*’ = 0.75			*E*’/*E*’’ = 1		
	ρ’’/ρ’	Ω_−_	Ω_0_	ε [%]	Ω_−_	Ω_0_	ε [%]	Ω_−_	Ω_0_	ε [%]	Ω_−_	Ω_0_	ε [%]
0.2	0.2	0.02830	0.02738	3.36	0.03771	0.03811	1.05	0.04438	0.04445	0.16	0.04978	0.04977	0.02
	0.5	0.02192	0.02120	3.40	0.02921	0.02951	1.02	0.03437	0.03443	0.17	0.03856	0.03855	0.03
	0.8	0.01853	0.01792	3.40	0.02469	0.02494	1.00	0.02905	0.02910	0.17	0.03259	0.03258	0.03
	1.0	0.01698	0.01642	3.41	0.02263	0.02286	1.01	0.02663	0.02667	0.15	0.02987	0.02986	0.03
0.5	0.2	0.02565	0.02662	3.64	0.03185	0.03173	0.38	0.03577	0.03570	0.20	0.03856	0.03855	0.03
	0.5	0.02294	0.02380	3.61	0.02849	0.02838	0.39	0.03199	0.03193	0.19	0.03449	0.03448	0.03
	0.8	0.02094	0.02172	3.59	0.02601	0.02590	0.42	0.02920	0.02914	0.21	0.03148	0.03148	0.00
	1.0	0.01987	0.02060	3.54	0.02467	0.02457	0.41	0.02770	0.02765	0.18	0.02987	0.02986	0.03
0.8	0.2	0.02908	0.02962	1.82	0.03055	0.03012	1.43	0.03168	0.03155	0.41	0.03259	0.03258	0.03
	0.5	0.02810	0.02861	1.78	0.02951	0.02910	1.41	0.03061	0.03048	0.00	0.03148	0.03148	0.00
	0.8	0.02720	0.02770	1.81	0.02857	0.02818	1.38	0.02963	0.02951	0.41	0.03048	0.03048	0.00
	1.0	0.02665	0.02714	1.81	0.02800	0.02761	1.41	0.02904	0.02891	0.45	0.02987	0.02986	0.03

The curves of the parameter of differences ε versus the ratios ρ″/ρ′ are shown in Figure A1.

Analysing results shown in Table A1 and Figure A1, it can be observed that:

1. Using the finite element method, only the lower frequency parameters, obtained in the framework of the tolerance model, can be compared and justified.
2. Differences between values of these parameters calculated using both methods are smaller than about 4% (3.64%) for the assumed geometrical and material parameters.
3. Smaller differences between obtained results (below 1.5%) are for these plate strips, which are described by the ratio *E*″/*E*′ ≥ 0.5 (*E*″/*E*′ = 0.5, 0.75, 1) and various values of the ratios—γ = 0.2, 0.5, 0.8; ρ″/ρ′ = 0.2, 0.5, 0.8, 1.
4. The biggest differences between calculated lower frequency parameters (above 3%) are for smaller values of the ratio *E*″/*E*′ (e.g., *E*″/*E*′ = 0.25) and some smaller values of the distribution parameter of material properties γ (e.g., γ = 0.2, 0.5).
